# Application of prolonged submental perforator flap to repair the postoperative defect of upper airway malignancy

**DOI:** 10.1007/s00405-023-08131-5

**Published:** 2023-08-02

**Authors:** Hongzhi Ma, Qi Zhong, Lizhen Hou, Ling Feng, Shizhi He, Meng Lian, Yanming Zhao, Ru Wang, Jugao Fang

**Affiliations:** 1 Department of Otolaryngology-Head and Neck Surgery, Beijing Tongren Hospital, Capital Medical University, 1 Dong Jiao Min Xiang, Eastern District, Beijing, 100730 China; 2 Key Laboratory of Otorhinolaryngology Head and Neck Surgery, Ministry of Education, Beijing Institute of Otorhinolaryngology, Beijing, 100730 China

**Keywords:** Extended submental perforator flap, Upper airway, Head and neck tumour, Repair, CT

## Abstract

**Objectives:**

To explore the feasibility of making a submental perforator flap distal to the connecting line between the mastoid and the sternoclavicular joint under the guidance of neck-enhanced CT and repairing the postoperative defect of upper airway malignancy.

**Materials and methods:**

This study retrospectively analysed 19 cases of upper airway malignant tumours treated in our department from January 2021 to September 2022, including 17 males and 2 females, aged 43–70 years.

**Site of lesions:**

15 cases were in the laryngopharynx, 2 cases in the nasal cavity and paranasal sinus and 2 cases on the soft palate. All the lesions were malignant and at stages T_2–4_N_0–2_M_0_.

**Surgical method:**

The extended submental perforator flap (size 22–15 × 6–7 cm) was prefabricated distal to the connecting line between the mastoid and the sternoclavicular joint. After tumour resection, the flap was used to repair the postoperative defect. Fifteen cases of laryngopharyngeal malignant tumours were repaired using the extended submental perforator flap with the vascular pedicle located on the opposite side of the tumour body. Two cases of nasal cavity and paranasal sinus tumours were repaired using the extended submental perforator flap combined with the temporalis muscle flap. The soft palate was completely removed in two patients with soft palate cancer and repaired using the folded extended submental perforator flap.

**Results:**

Before the surgery, the reflux vein was observed by neck-enhanced CT, including 12 cases returning to the internal jugular vein and 7 cases to the external jugular vein. All 19 cases in which flaps were used survived, and 1 case had a postoperative infection. All the patients had nasal feeding removed after surgery. The tracheal cannula was removed from the patients with laryngeal preservation, and the pronunciation was satisfactory. Among them, patients with soft palate cancer repair had mild nasal reflux symptoms with smooth breathing. During the follow-up period of 4–24 months, 18 patients had no tumour recurrence or metastasis, and 1 patient had cervical lymph node metastasis.

**Conclusions:**

This study highlights the use of a submental perforator flap distal to the connecting line between the mastoid and the sternoclavicular joint to repair postoperative defects for upper airway malignancy as an innovative surgical approach that provides more tissue and good arteriovenous blood supply to adjacent sites. This method has high clinical value and provides an effective option for repairing postoperative defects of upper airway malignancy.

**Supplementary Information:**

The online version contains supplementary material available at 10.1007/s00405-023-08131-5.

## Introduction

The upper airway is anatomically divided into three parts, i.e., the nose, pharynx and larynx, which are all important organs of the head and neck [1]. The upper airway is deep and hidden; as such, patients with smaller lesions may not have obvious symptoms, with most of these in mid-to-terminal stages when found. Surgery is the main treatment for malignant upper airway tumours. Particularly in patients with middle and late-stage tumours, the defect will be large after resection and require tissue valve repair and reconstruction [2, 3]. In the past, there have been reports of repair using the pectoralis major, supraclavicular flap, gastric pull-up, temporalis and submental and free tissue flaps, each of which has advantages and disadvantages [4–10]. Free flap has forearm flap, lateral arm flap and lower medial leg fasciocutaneous flap and others, with moderate thickness. The tissue volume is fully adjustable, and the reconstruction effect is good. However, a free tissue flap involves vascular anastomosis, which requires specific anastomotic vascular conditions and specialised anastomosis skills.

Many patients with malignant upper airway tumours are elderly, long-time smokers, maintain a poor diet and suffer from vascular sclerosis and generally poor vascular conditions. These patients are at high risk of vascular crisis during vascular anastomosis. Once necrosis occurs in the flap, remediation is difficult [4–7].

In 1993, Martin et al. first reported the anatomical and clinical characteristics of the submental flap [8]. The flap is of moderate thickness and, if used to repair upper airway defects, the reconstructed functioning will be closest to the original physiological state. The conventional submental flap was taken from the medial side of the mandibular angle, and submental flaps are mainly used for the reconstruction of ipsilateral defects of the head, neck, and oral cavity. Limited by length, however, it cannot repair the nasal cavity, soft palate, pharynx or other distant areas. In addition, because there are many variations in the submental vein returning to the proximal cardiac vein, the abnormal venous return also appears in the perioperative period of the mental flap Vascular crisis is the main cause of flap stasis necrosis [9–11]. Preoperative enhanced computed tomography (CT) is an effective method for predicting the reflux vein and its walking in the submental flap, which will greatly improve the survival rate of the submental perforator flap [12].

The prolonged submental perforator flap has a moderate thickness, simple operation, reliable blood supply, flexible rotation, is close to typical head and neck defect sites, and can be removed in the same surgical procedure as the tumour. The donor site scarring can easily be hidden behind the mandible with good aesthetic effect and speech and swallowing function repair to the throat is satisfactory. Based on previous surgical experience, we attempted to create an extended submental perforator flap distal to the connecting line between the mastoid and the sternoclavicular joint to repair postoperative defects in the upper airway in the nasal cavity, soft palate and contralateral larynx and pharynx, as well as other relatively distant areas with less trauma and straightforward surgery.

## Data and methods

### Clinical data

**Fig. 1:**
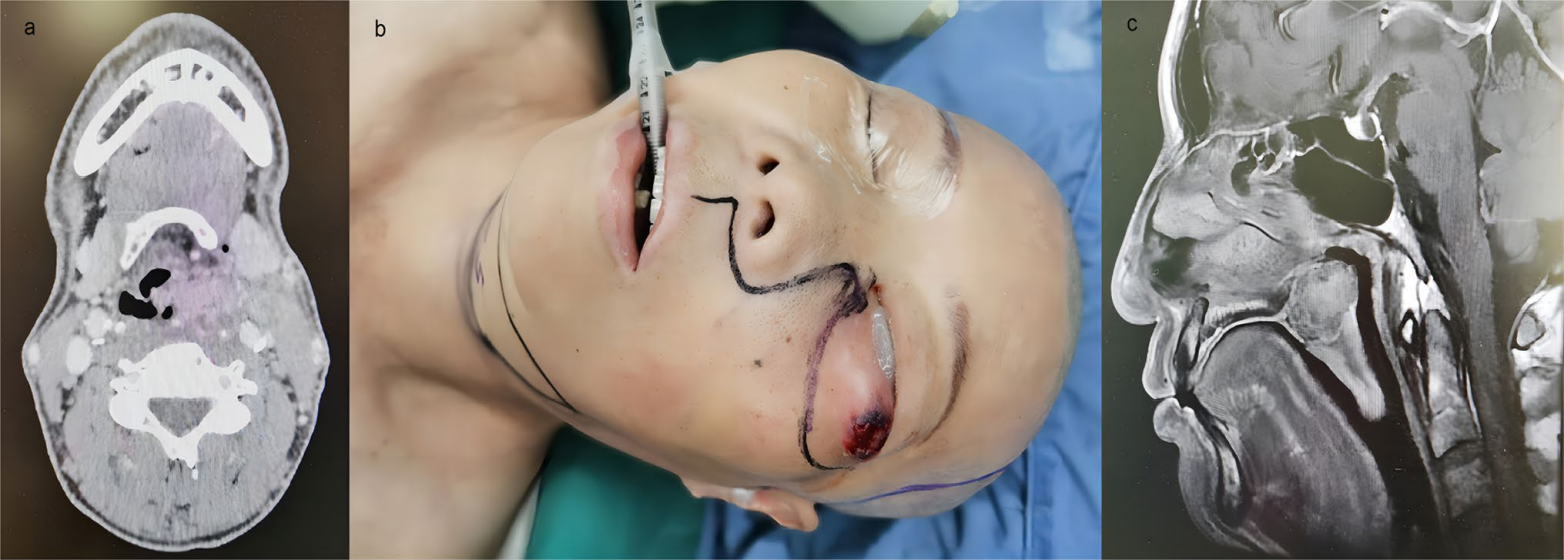
**a** Laryngopharyngeal lesions invaded the reflux vein of the ipsilateral submental flap. **b** Lesions of the maxillary sinus invaded the skin of the ipsilateral lower eyelid region. **c** Soft palate lesions

From January 2021 to September 2022, 19 patients were treated in our department for upper airway malignancies, including 17 males and 2 females, aged 43–70 years. The lesion sites were as follows: 15 cases were in the larynx and pharynx, 2 in the maxillary sinus and 2 cases in the soft palate (see Fig. 1a–c), including 16 cases of squamous cell carcinoma, 1 adenoid cystic carcinoma, 1 neuroendocrine carcinoma and 1 case of pleomorphic adenocarcinoma. The clinical staging of malignant lesions before treatment (according to AJCC 2017 criteria) were as follows: T_2–4_N_0–2_M_0_, including 6 cases of T_2_, 5 of T_3_, 8 of T_4_, 6 of N_0_, 8 of N_1_ and 5 cases of N_2_. Seven patients did not achieve large PR (more than 70% reduction) after 2 cycles of induction chemotherapy [13]. After MDT discussion and doctor–patient communication, we decided to conduct a surgical intervention. Enhanced CT/MRI of the lesion site, neck-enhanced CT, neck lymph node ultrasound, a systemic general status assessment, systemic tumour metastasis evaluation and submental beard distribution assessment (in male patients) were performed before surgery. Preoperative examination indicated that two patients with laryngopharyngeal carcinoma were accompanied by early oesophageal carcinoma (for whom subsequent resection under gastroscopy 1 month after surgery for laryngopharyngeal carcinoma was performed).

The patients were in good general health, with an ECOG score of 0 or 1 point. All of the 19 patients underwent surgery under general anaesthesia. The surgery method was as follows: radical surgery of the primary tumour was performed at the same time as extending the submental perforator flap for repairing the defect after tumour resection. Fifteen cases of laryngopharyngeal carcinoma were repaired using the extended submental perforator flap with the vascular pedicle contralateral to the main body of the tumour; 11 patients underwent throat preservation surgery, and 4 patients underwent extended total laryngectomy. Postoperative defects of the nasal cavity were repaired. In addition, the paranasal sinus of 2 maxillary sinus cancer cases was repaired using the extended submental perforator flap combined with a temporalis muscle flap. One patient with malignancy underwent orbital content resection, and the other patient had thin temporal muscles. The medial soft palate of the pterygoid process was completely removed in two cancer cases and the extended submental perforator flap was used to repair the oral and nasal surfaces of the soft palate (see Table 1).

**Table 1:** Repair of upper airway defect with extended mental flap after resection of malignant tumors in 19 cases

Case number	Gender	Age	Tumor location	Stage	Lesion body	Operation method	Prediction of the reflux vein of the submental flap by CT	Reflux vein of the submental flap	Size of the submental flap	Distance from the distal end to the mandibular angle	Survival status of the submental flap	Preoperative chemotherapy/radiotherapy	Postoperative follow-up	Postoperative condition (VHI-10 score/GUSS score)
1	Male	47	Squamous cell carcinoma	(T4N2bM0)	Laryngopharynx	A	External jugular vein	External jugular vein	15 × 6 cm	5 cm	Survive	No/radiotherapy	17 months	Removal nasal feeding and tracheal cannula (1/20)
2	Male	62	Squamous cell carcinoma	(T2N0M0)	Laryngopharynx	A	Internal jugular vein	Internal jugular vein	15 × 6 cm	4 cm	Survive	No/radiotherapy	15 months	Removal nasal feeding and tracheal cannula (3/18)
3	Male	56	Squamous cell carcinoma	(T4N2aM0)	Laryngopharynx	A	Internal jugular vein	Internal jugular vein	15 × 6 cm	4 cm	Survive	2 cycles/radiotherapy	7 months	Removal nasal feeding and tracheal cannula (7/17)
4	Male	55	Squamous cell carcinoma	(T4N1M0)	Laryngopharynx	A	Internal jugular vein	Internal jugular vein	15 × 6 cm	4 cm	Survive	2 cycles/radiotherapy	8 months	Removal nasal feeding and tracheal cannula (4/15)
5	Male	49	Squamous cell carcinoma	(T2N2bM0)	Laryngopharynx	A	External jugular vein	External jugular vein	15 × 6 cm	5 cm	Survive	2 cycles/radiotherapy	9 months	Removal nasal feeding and tracheal cannula (1/20)
6	Male	52	Adenoid cystic carcinoma	(T2N0M0)	Laryngopharynx	B	Internal jugular vein	Internal jugular vein	15 × 6 cm	4 cm	Survive	No/radiotherapy	10 months	Removal nasal feeding/carrying tube (no/20)
7	Male	70	Squamous cell carcinoma	(T3N1M0)	Laryngopharynx	C	External jugular vein	External jugular vein	20 × 6 cm	3 cm	Survive	2 cycles/radiotherapy	5 months	Removal nasal feeding and tracheal cannula (0/19)
8	Male	68	Squamous cell carcinoma	(T3N1M0)	Laryngopharynx	A	External jugular vein	External jugular vein	15 × 6 cm	4 cm	Survive	No/radiotherapy	15 months	Removal nasal feeding and tracheal cannula (4/19)
9	Male	55	Squamous cell carcinoma	(T3N1M0)	Laryngopharynx	A	External jugular vein	External jugular vein	15 × 6 cm	4 cm	Survive	2 cycles/radiotherapy	9 months	Removal nasal feeding and tracheal cannula (3/18)
10	Male	43	Squamous cell carcinoma	(T4N1M0)	Laryngopharynx	A	Internal jugular vein	Internal jugular vein	15 × 6 cm	5 cm	Survive	No/radiotherapy	8 months	Removal nasal feeding and tracheal cannula (3/19)
11	Male	69	Squamous cell carcinoma	(T3N1M0)	Laryngopharynx	A	Internal jugular vein	Internal jugular vein	15 × 6 cm	4 cm	Survive	2 cycles/radiotherapy	16 months	Removal nasal feeding and tracheal cannula (4/17)
12	Female	56	Neuroendocrine carcinoma	(T3N2M0)	Laryngopharynx	B	Internal jugular vein	Internal jugular vein	15 × 6 cm	3 cm	Survive	2 cycles/radiotherapy	7 months	Removal nasal feeding/carrying tube (no/20)
13	Male	66	Squamous cell carcinoma	(T2N2M0)	Laryngopharynx	A	Internal jugular vein	Internal jugular vein	15 × 6 cm	4 cm	Survive	No/radiotherapy	18 months	Removal nasal feeding and tracheal cannula (2/18)
14	Male	56	Squamous cell carcinoma	(T4N1M0)	Laryngopharynx	B	External jugular vein	External jugular vein	20 × 7 cm	5 cm	Survive	No/radiotherapy	8 months	Removal nasal feeding/carrying tube (no/20)
15	Male	60	Squamous cell carcinoma	(T4N1M0)	Laryngopharynx	B	Internal jugular vein	Internal jugular vein	20 × 7 cm	4 cm	Infection/survive	No/radiotherapy	5 months	Removal nasal feeding/carrying tube (no/20)
16	Male	65	Squamous cell carcinoma	(T2N0M0)	Soft palate	D	Internal jugular vein	Internal jugular vein	20 × 6 cm	4 cm	Survive	No/radiotherapy	22 months	Removal nasal feeding and tracheal cannula (2/17)
17	Male	49	Pleomorphic adenocarcinoma	(T2N0M0)	Soft palate	D	External jugular vein	External jugular vein	20 × 6 cm	5 cm	Survive	No/radiotherapy	6 months	Removal nasal feeding and tracheal cannula (2/18)
18	Male	68	Squamous cell carcinoma	(T4N0M0)	Maxillary sinus	E	Internal jugular vein	Internal jugular vein	22 × 7 cm	5 cm	Survive	No/radiotherapy	9 months	Removal nasal feeding and tracheal cannula (1/19)
19	Female	55	Squamous cell carcinoma	(T4N0M0)	Maxillary sinus	F	Internal jugular vein	Internal jugular vein	20 × 6 cm	4 cm	Survive	No/radiotherapy	6 months	Removal nasal feeding and tracheal cannula (1/19)

A: Partial larynx resection + laryngopharyngeal mass resection + neck dissection + contralateral submental flap repair

B: Total laryngectomy + laryngopharyngeal mass resection + neck dissection + contralateral submental flap repair

C: laryngopharyngeal mass resection + neck dissection + contralateral submental flap repair

D: Soft palate resection + neck dissection + submental flap repair

E: extended resection of the maxilla + resection of orbital contents + repair with submental flap and temporalis muscle flap

F: extended resection of the maxilla + repair with submental flap and temporalis muscle flap

This study was approved by the Institutional Ethics Committee of theBeijing Tongren Hospital, Capital Medical University (ethics nr: TREC2022-KY018.R1), and written informed consent was obtained from all participants for their inclusion in the research.

### Surgical method

#### Predicting the reflux vein of the submental flap before surgery

**Fig. 2:**
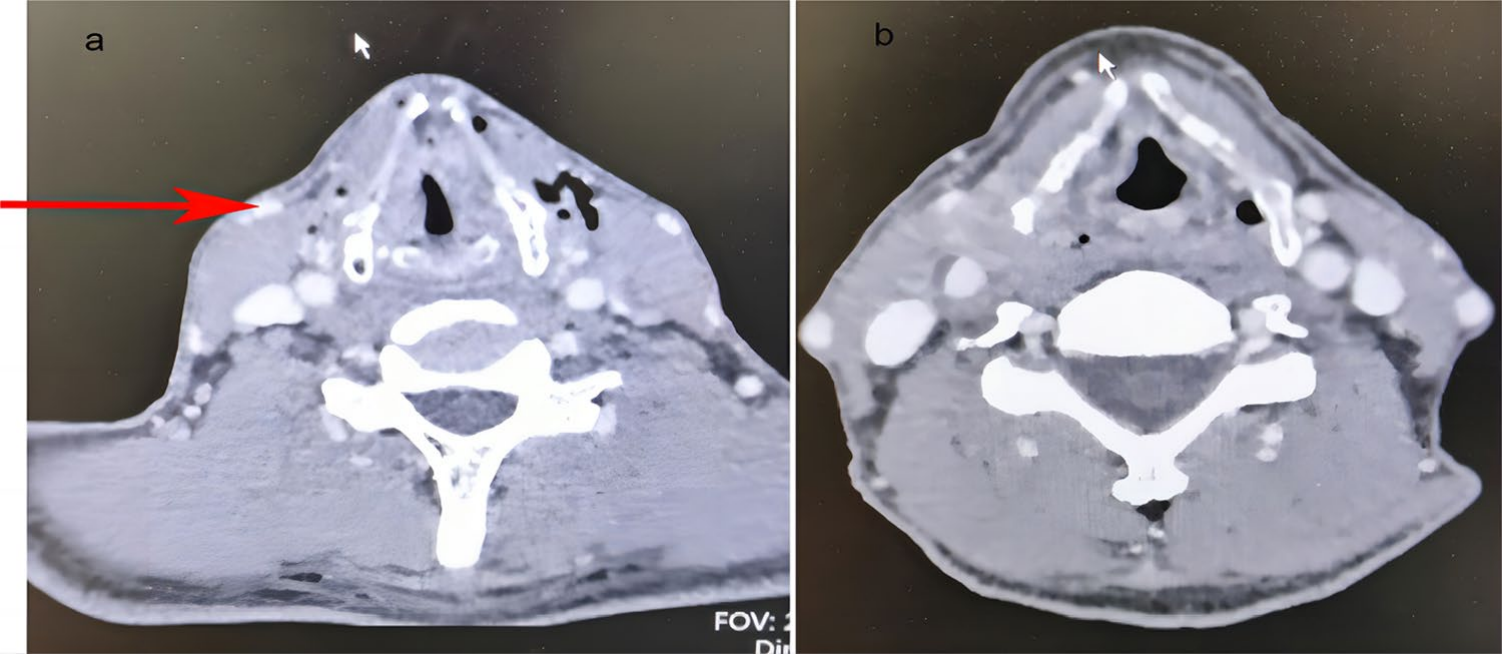
**a** Reflux vein of the submental flap enters into the external jugular vein through the surface of the submandibular gland and the anterior edge of the sternocleidomastoid muscle at the level of the thyroid cartilage. **b** Reflux vein of the submental flap enters into the internal jugular vein through the surface of the submandibular gland and the anterior edge of the sternocleidomastoid muscle at the level of the thyroid cartilage

All patients underwent a high-resolution thin slice enhanced CT scan of the neck before surgery. The baseline scan of the lower orbital line was performed by spiral scanning using a pitch of 0.8–1.0. The soft tissue algorithm was reconstructed, and the thickness of the reconstructed layer was 1.0 mm. The iodine contrast agent concentration was 300 mg/ml, the dosage was 1.0–1.5 ml/kg, the injection flow rate was 2.5–3.5 ml/s, and the enhanced scanning time was 50–60 s after the contrast agent was injected. Using a CT venous-phase continuous-level scan, the neck lymph metastasis was observed and the venous reflux and walking of the submental area tissue were predicted (see Fig. 2a, b).

#### Surgery procedure

① Preparation of the extended submental perforator flap.

**Fig. 3:**
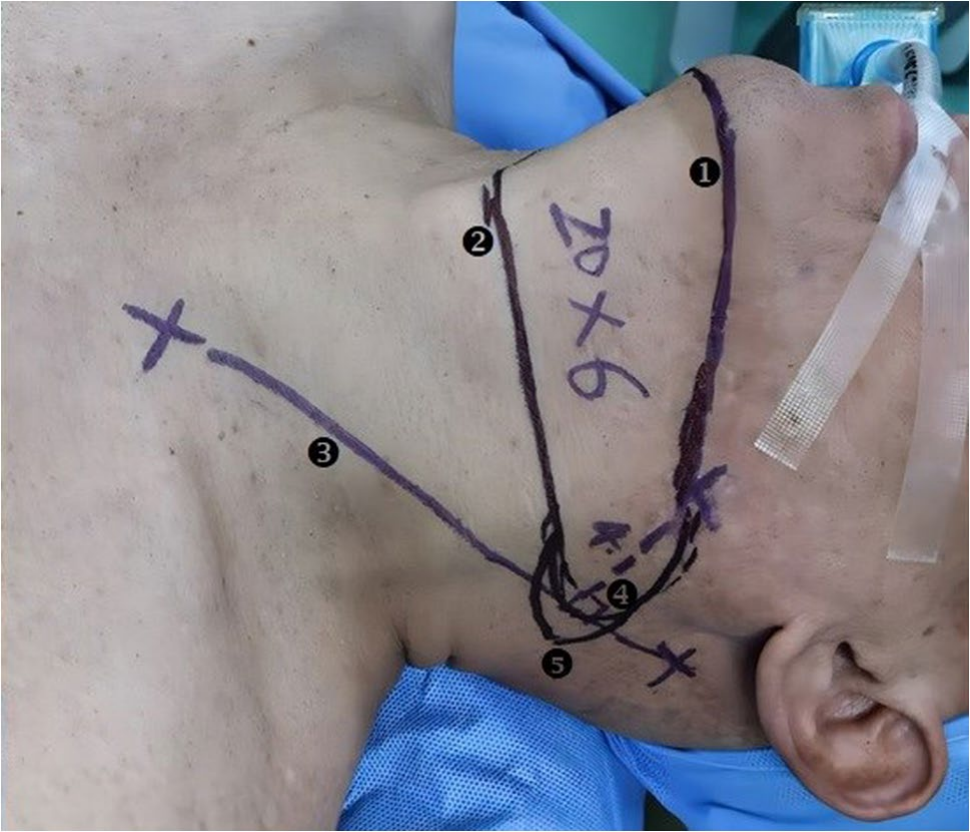
Design of extended submental perforator flap. 1. The upper margin of the flap is 0.5 cm away from the lower margin of the mandible; 2. The lower margin of the flap is located at the upper margin of the anterior median of the cervical cartilage; 3. The connecting line between mastoid and the sternoclavicular joint. 4.The intersection of perpendicular line form the mandibular angle to the connecting line between mastoid and the sternoclavicular joint. 5.The contralateral side of the pedicle is 1 cm outside the intersection of perpendicular line form the mandibular angle to the connecting line between mastoid and the sternoclavicular joint

The flap design was as follows: 0.5 cm from the lower edge of the mandible, the superior margin of the flap was designed along the inferior border of the mandible. The border of the pedicle side was extended to the lateral side of the submandibular gland. The contralateral side of the pedicle was 1 cm outside the intersection of the perpendicular line from the mandibular angle to the connecting line between the mastoid and the sternoclavicular joint. The lower margin of the flap was located at the upper margin of the anterior median of the cervical cartilage. The size of the flap was 22–15 × 6–7 cm (see Fig. 3).

**Fig. 4:**
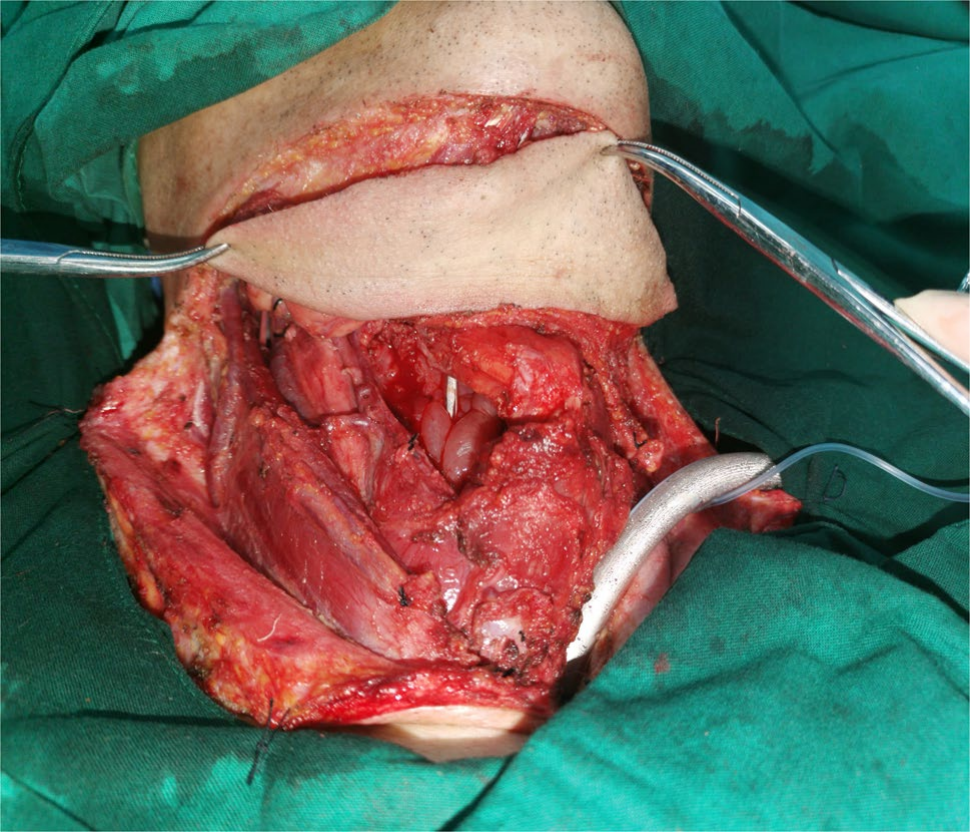
Preset of extended submental perforator flap

Based on the flap design, the skin, subcutaneous tissue and latissimus dorsi muscle of the neck were cut. On the surface of the facial artery, at the anterior margin of the masseter muscle, the facial nerve marginal mandibular branch was dissected and protected. The anterior belly of the pedunculated digastric muscle was severed below the mandible. Care was taken to avoid separating the anterior belly of the digastric muscle from the submental flap. The submental artery between the upper edge of the submandibular gland and the lower edge of the mandible was located and protected from the submental flap side. Based on the preoperative CT prediction, the reflux vein was confirmed and was retained in the submental flap to protect it, as well as the superior vein. Based on the relationship among the submandibular gland, the submental artery, the reflux vein and local lymph nodes, the submandibular gland can be removed or retained at the root of the submental flap. The flap was then prepared for use (see Fig. 4).

② Radical surgery for the primary tumour.

The neck lymph node dissection proceeded as follows. According to the preoperative cervical lymph node ultrasound and neck CT, neck lymph node dissection was performed, and caution was taken to protect the blood supply of the prefabricated submental flap during the operation.

The process for laryngopharyngeal mass resection + partial laryngectomy/total laryngectomy was as follows. The epiglottic vallecula or lateral wall approach to the pharynx on the side of the lesion was adopted. According to the extent of lesion invasion, the tumour was removed using a 2 cm safety margin under direct observation (including part of the larynx as appropriate). Total laryngeal resection was performed if the lesion invaded the posterior commissure, the postcricoid region or the bilateral vocal compartment bands.

The process for extended maxillary resection was as follows. One malignant tumour case involved orbital content; here, the maxilla and orbital contents on the affected side were removed. Another malignancy in a female patient involved the lower eyelid and facial skin, and the temporal muscle was thin; in this case, the mandible and the lower eyelid and local facial skin of the affected side were removed.

Excision of the soft palate tumour proceeded as follows. In two cases, the main body of the lesion was located in the middle of the soft palate. The medial soft palate of the pterygoid hamulus and part of the posterior bones of the hard palate were removed with an adequate safety margin.

③ Using the extended submental perforator flap to repair the defect after tumour resection.

Blood supply at the distal end of the submental flap was confirmed. The criteria for good blood supply to the flap were as follows. The epidermis of the most distal 2 × 0.5 cm of the flap was removed and dermis bleeding was observed. The flap had a good blood supply with diffuse blood infiltration. If the blood supply is poor, the distal end will be removed and re-evaluated until the blood supply is good, and the relevant data will be assessed.

**Fig. 5:**
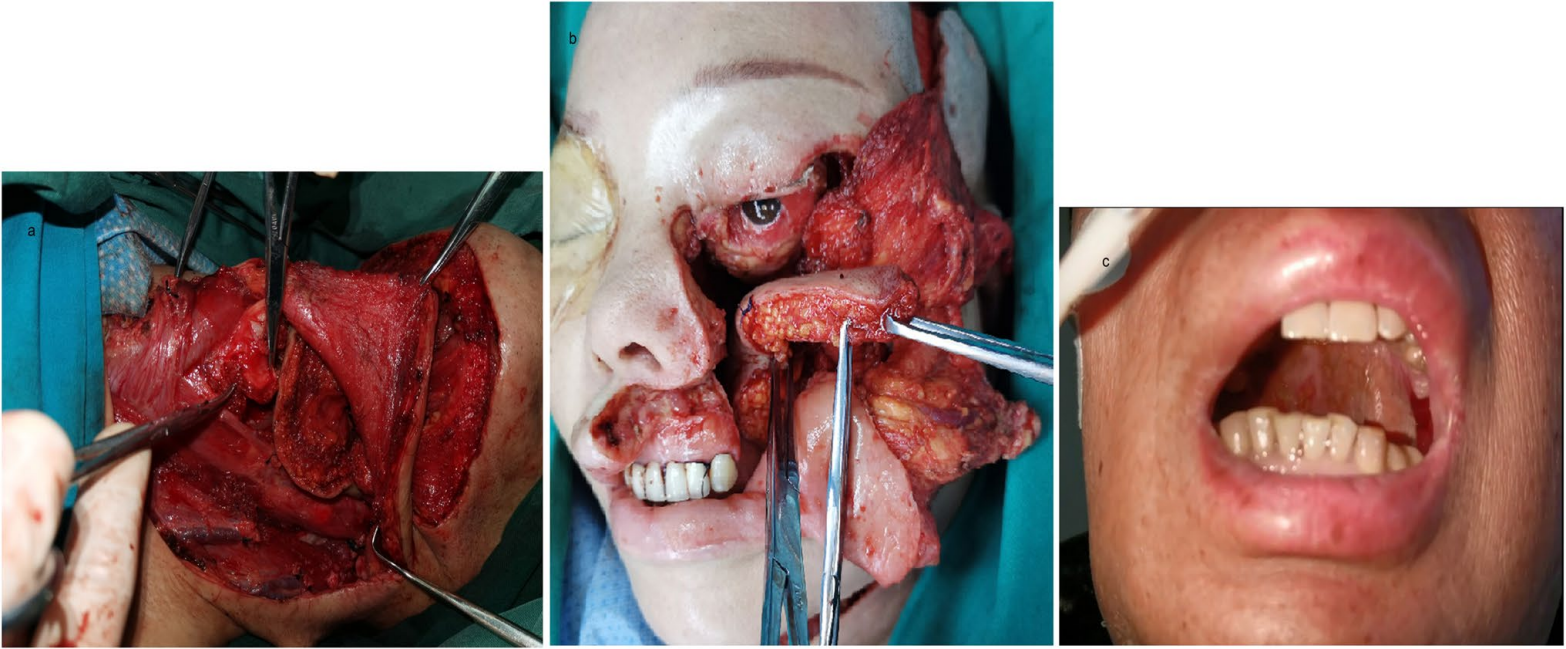
**a** Remodeling of piriform fossa and repair defect with contralateral extended submental flap (laryngopharyngeal carcinoma). **b** Repair hard palate area + lateral wall of nasal cavity + lower eyelid with extended submental perforator flap combined with temporal muscle (cancer of maxillary sinus). **c** Repair of nasal and oral surfaces of soft palate after extended submental perforator flap folding (soft palate cancer)

The repair of postoperative defects in cases of laryngopharyngeal tumours proceeded as follows. The skin surface of the submental flap is arranged to face the side of the pharyngeal cavity, and the roots of the submental flap are protected (torsion below 180°). For patients with partial laryngopharyngectomy and partial laryngectomy, the long axis of the submental flap was arranged parallel to the lower edge of the mandible. The flap crossed the upper part of the larynx between the hyoid bone and the upper margin of the thyroid cartilage of the unaffected side (the pre-epiglottic space) and was transferred to the affected side. The submental flap was smoothed and adjusted to ensure that it had no tension and blood supply was not affected. The distal end of the mental flap was appropriately turned to reconstruct the pyriform fossa. During the suture process, for excess near-end skin of the submental flap, the epidermis was removed, and care was taken to protect the subcutaneous tissue and subcutaneous blood supply (see Fig. 5a). For patients undergoing total laryngectomy, the submental flap was directly sutured to the residual posterior wall of the laryngopharynx to close it (see Supplementary Image 1).

Postoperative maxillary defect repair proceeded as follows. In the parapharyngeal space under the jaw, the pre-set submental flap was delivered through the medial mandibular tunnel to the area of the maxillary defect. For one case with maxilla and orbital content resection, the extended submental perforator flap was used to repair the hard palate and the lateral nasal wall, and the temporal muscle was filled into the facial skin and orbital content area. For another case with thin temporal muscle and an absent lower eyelid, the extended submental flap was used to repair the hard palate surface, nasal surface and lower eyelid and facial skin, and the temporalis muscle was filled into the maxillary area (see Fig. 5b and Supplementary Image 2).

To repair the defect after resection of the soft palate, in the parapharyngeal space of the submandibular gland, through the medial part of the mandible to the front area of the tonsil, the extended submental flap was set parallel to the posterior edge of the hard palate, the skin surface was tiled towards the mouth, and the distal end of the flap was folded back to form the nasal cavity surface. Subsequently, the excess epidermis was removed, and the submental flap was stitched and fixed to reshape the soft palate (see Fig. 5c and Supplementary Image 3).

④ Placing the drainage and closing the surgical cavity.

After adequate haemostasis, a negative pressure drain was placed in the neck surgical area. The platysma, subcutaneous tissue and skin were sutured and protected by sterile gauze. The patient's head was kept in a forward-facing position and pressure was prohibited in the root of the neck flap.

## Results

The reflux vein of the submental flap was explored intraoperatively, including 12 involving the internal jugular vein and 7 cases including the external jugular vein. All the cases matched with the preoperative neck enhanced CT prediction, with a compliance rate of 100%. There were no differences in the blood vessels and blood supply of the submental flap during the surgery between patients undergoing preoperative induction chemotherapy and those without induction chemotherapy. All the flaps had survived 2 weeks after surgery. The resection margins of the specimens of all patients were negative, the bleeding volume was 200–500 ml and the average operation time was 7.5 h. Only one patient developed a local effusion infection and was discharged after conservative treatment with a dressing change 10–14 days after surgery.

**Fig. 6:**
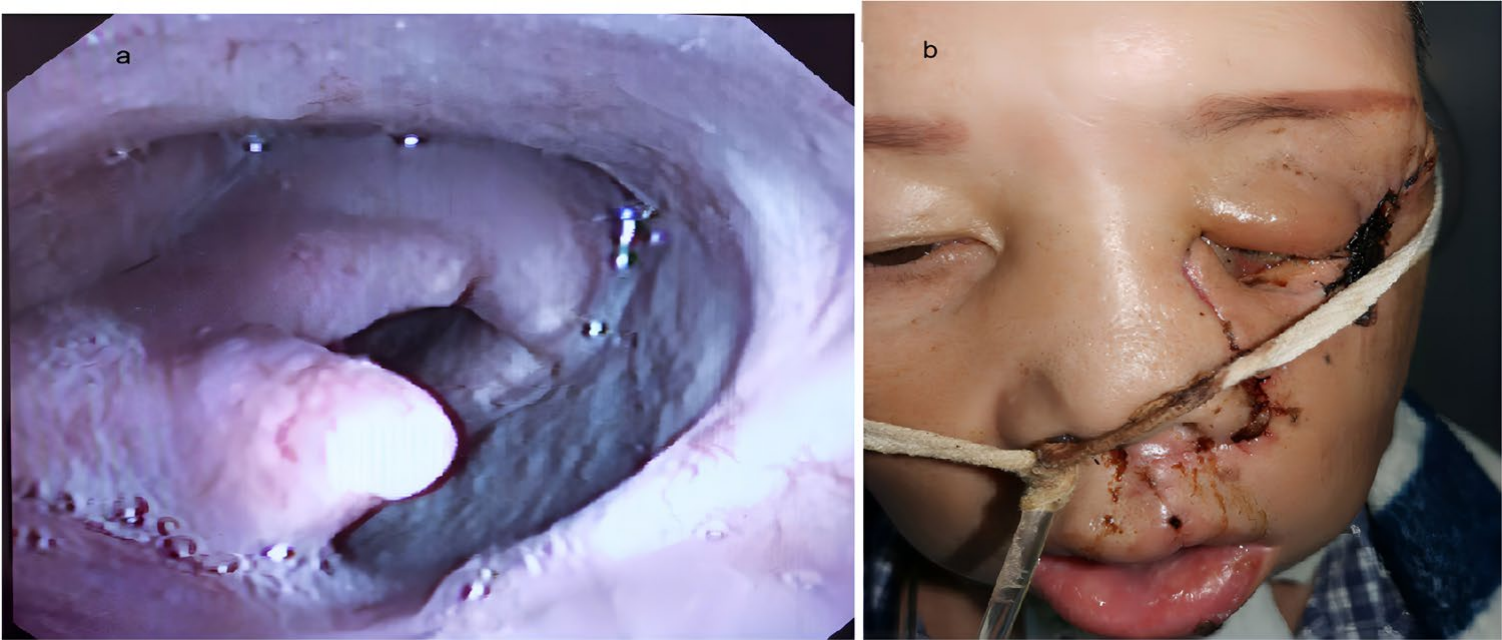
**a** One year after postoperative repair for laryngopharyngeal carcinoma with contralateral extended submental perforator flap. **b** 1 week after repair of maxillary sinus cancer with extended submental perforator flap

By January 2023, the follow-up period ranged from 4 to 24 months. The average follow-up period was 18 months, and 16 patients underwent postoperative adjuvant radiotherapy. Following surgery, all patients had smooth breathing. Except for 4 patients who underwent total laryngectomy, all had continuous tube plugging 2 weeks after surgery, with good pronunciation quality. The VHI-10 score of the subjective evaluation of voice was 0–7. The GUSS score for swallowing function was 15–20. Except for 1 case, whose nasal feeding was removed after 3 months due to severe underlying cardiopulmonary disease, the nasal feeding tubes of the remaining 18 patients were removed within 1–4 weeks after surgery. There was no primary site recurrence during follow-up in 18 patients. One case developed neck metastasis 5 months after surgery (2 months after radiotherapy). One patient developed lower oesophageal squamous cell carcinoma 1 year after surgery and was given chemoradiation therapy. Pulmonary metastases were detected 1 year after surgery in a patient with adenoid cystic carcinoma who received chemotherapy and survived with the tumour (see Fig. 6a, b).

## Discussion

The submental flap was adjacent to the primary lesion of the upper airway. There is no important organ structure around the submental flap, which makes the flap-harvesting operation simple and easy to perform, and secondary damage to the flap area can be ignored. The submental flap is of moderate thickness and easy to fold. It is used to repair defects in upper airway parts, such as the nasal cavity, oral cavity and throat, which is conducive to functional recovery [14, 15]. Presently, the length of the submental flap is typically located in the submental area between the bilateral mandibular angles, which can be used in the repair of the floor of the mouth, tongue, tonsil area and larynx–pharynx defects on the root side. For nasal cavity and paranasal sinus defects, as well as for use in the soft palate and throat on the opposite side of the root of the submental flap, the conventional submental flap taken between the bilateral mandibular angle is limited by length and, as such, cannot be used in repair processes [16, 17].

In recent years, other studies on distal flap harvesting of the submental flap have been conducted, e.g., for repairing oral and maxillofacial defects distal to the donor site by the vascular pedicle of the extended submental artery perforator flap [18]. In our work, we studied the limit of the distal end of the submental flap and found that, on the medial side of the connecting line between the mastoid and sternoclavicular joint this flap had good blood supply, without postoperative necrosis. In 19 cases in this study, the upper end of the submental flap was along the lower edge of the mandible; the widest area of the lower end was the upper edge of the thyroid cartilage, and the root side was on the outside of the submandibular gland. The distal design was 1 cm lateral from the intersection between the vertical line from the mandibular angle and the connecting line between the mastoid and the sternoclavicular joint. After harvesting the flap, the blood supply to the most distal end of the flap was observed. If the blood supply was poor, the flap was cut with scissors until the blood supply improved, and the distance between the most distal end of the flap with good blood supply and the mandibular angle was measured.

Likely influenced by age, gender, body weight and neck circumference, the distance between the distal end of the flap and the mandibular angle was between 3 and 5 cm, with individual differences. If the distance from the mandibular angle is the standard, accurate preoperative prediction of the distal end of the extended submental perforator flap cannot be made. We also found that the blood supply in the submental flap of the connecting line between the mastoid and the sternoclavicular joint and its medial side was good and without postoperative necrosis, while the submental flap outside the connecting line may have had poor blood supply. With the connecting line between the mastoid and the sternoclavicular joint as the distal boundary of the extended submental perforator flap, it is reliable as a position and not affected by individual differences. It can increase the conventional submental flap area distal to the mandibular angle by 8–15 cm^2^.

Continuous thin-layer scanning of neck-enhanced CT was used to accurately predict the reflux vein and its walking of the submental flap, which can help to guide the protection of this vein during surgery [9, 19]. The literature suggests that the reflux veins of the submental flap are generally the external jugular or internal jugular veins. Postoperative reflux venous disturbance is one of the main causes of submental flap failure [20]. The reflux vein of the submental flap, as suggested by preoperative CT, is fully consistent with the actual reflux vein of the submental flap during surgery; this helps to effectively overcome the difficulty of protecting the reflux vein in the harvesting of the mental flap, and greatly improve the speed and survival rate of the submental flap harvesting.

We found that the extended submental perforator flap has a significant role in the repair of larger postoperative defects related to upper airway malignancy, particularly in functional reconstruction. Related reports show that if there is a large defect in the site in the upper airway away from the submental region, the conventional submental flap will not be able to fully cover such a defect [16, 17]. The extended submental perforator flap increases the size of the tumor area to 3–5 × 3 cm compared with the conventional submental flap, which can make up for the defects of the latter and complete the full structural remodelling and functional reconstruction of the above areas.

In our study of using the extended submental flap in the repair of contralateral laryngopharyngeal carcinoma, our team first proposed using the submental flap located on the contralateral side of the lesion body to repair the postoperative defect of laryngopharyngeal carcinoma and to reconstruct the larynx, laryngopharynx and upper oesophagus [21, 22]. In the repair of laryngopharyngeal cancer, compared with using the submental flap with a pedicle on the same side of the lesion, using the submental flap with a pedicle on the opposite side overcomes the adverse effects of dissecting an enlarged lymph node on the neck on the submental flap, particularly on the reflux vein, and has more advantages and positive features [23]. However, for a defect at the bottom of the piriform fossa and the upper end of the oesophagus, in most cases, the conventional contralateral submental flap can only take tension sutures due to its length limitation, and there is insufficient tissue for piriform fossa remodelling [24, 25]. Our study suggests that the extended submental perforator flap may be sufficient for repairing a defect in the contralateral piriform fossa and the upper oesophagus and for use in anatomical reconstruction. Doing so effectively avoids the risk of food inhalation into the airway using direct sutures (no piriform fossa reconstruction). Concurrently, enough tissue was obtained to overlap with the upper oesophagus connected to the larynx and pharynx, minimising the occurrence of postoperative oesophageal entrance stenosis. The extended submental perforator flap achieved tension-free suturing and also greatly reduced the risk of infection and rupture in the postoperative suture area.

Studies have also been conducted on the repair of the extended submental perforator flap in the lateral wall of the nasal cavity. A conventional flap can repair the hard palate but has difficulty reaching the lateral wall of the nasal cavity [26]. The two cases involving the postoperative repair process of the maxillary sinus in our study indicated that the extended submental perforator flap could be used to reconstruct the lateral wall of the nasal cavity after maxillary surgery and could even reconstruct the inferior orbital wall and partially repair facial skin defects (see Fig. 6b). It is, however, less likely to have nasal adhesion compared with temporalis muscle repair and also increases the amount of tissue needed for local repair. The extended submental perforator flap is a very innovative repair approach for patients with thin temporalis/orbital content that is removed at the same time and who are not suitable candidates for free flap repair.

Finally, research has been conducted on the extended submental perforator flap for functional reconstruction of the soft palate. Soft palate cancer is relatively rare, and the repair after soft palate resection is more difficult compared with upper airway defect repairs. The conventional submental flap can reach the soft palate; however, due to an insufficient amount of distal tissue, the soft palate can only be repaired on a single side and soft palate remodelling falls short; in addition, nasal reflux will be heavy after surgery. In addition, for single-side repair, the wound tissue (without skin) is exposed to the airway, making it prone to infection and flap necrosis [27, 28]. Both of our patients underwent bilateral soft palate resection on the medial side of the pterygoid hamulus, and the extended flap provided adequate tissue for autologous folded double-sided (oral and nasal) soft palate repair. Both of our patients had smooth postoperative nasal breathing. At 22 months after surgery, one patient had mild nasal reflux with oral feeding. Half a year after surgery, another patient had no nasal reflux with oral feeding.

The shortcomings of this study’s participant group are the small number of cases (19), short follow-up time (4–24 months) and the long-term oncology effect, which requires further observation. We focused on the local repair method, however, which is sufficient to illustrate the feasibility of a new surgical method to repair the postoperative defect of upper airway malignancy using the extended submental perforator flap distal to the connecting line between the mastoid and sternoclavicular joint. In this group, 17 patients were male without a dense submental beard and had submental perforator flap suitable for repair. In addition, among East Asian men, particularly in the middle-aged and elderly population, the submental beard is sparser, and the submental flap is suitable for repairing pharyngeal and laryngeal defects. However, for male patients with a dense submental beard, the repair method should be carefully selected. Secondary hair removal treatment can also be selected in these cases. The extended submental perforator flap has more indications than the conventional submental flap, but the former cannot be prolonged without limitations. In these cases, other repair methods can be selected when the repair conditions are not met.

In conclusion, the extended submental perforated flap, distal to the connecting line between the mastoid and the sternoclavicular joint, was used to repair the postoperative defect in cases of upper airway malignancy, with good blood supply, moderate thickness and reduced trauma. Compared with the conventional submental flap, the tissue volume is greater, the pedicle is longer, and the available repair area is larger. This method can reach areas that in the past could not be achieved using the conventional submental flap and is a reliable and effective approach for effecting repair after upper airway tumour resection and is, accordingly, worth promoting. Under the guidance of preoperative CT, the reflux vein and its walking of the submental flap were predicted, thereby solving difficulties in the preparation of the submental flap and ensuring the survival rate of the submental flap.

### Supplementary Information

Below is the link to the electronic supplementary material.

Supplementary file1 Figure 1a Preset chin flap (JPG 2800 KB)

Supplementary file2 Figure 1b Lateral incision (JPG 3531 KB)

Supplementary file3 Figure 1c T-shaped incision (JPG 2255 KB)

Supplementary file4 Figure 1d Resected lesion (piriform fossa carcinoma) (JPG 3512 KB)

Supplementary file5 Figure 1e Contralateral mental flap was used to repair the postoperative defect of the piriform fossa carcinoma (JPG 2574 KB)

Supplementary file6 Figure 1f Contralateral mental flap repair wound after total laryngectomy (retrocircumferential carcinoma) (JPG 3790 KB)

Supplementary file7 Figure 1g Contralateral mental flap was repaired for posterior pharyngeal wall carcinoma, and the affected side of the piriform fossa was reconstructed (JPG 2469 KB)

Supplementary file8 Figure 2a Extended submental perforator flap to repair the defect after tumor resection (JPG 3149 KB)

Supplementary file9 Figure 2b Repair hard palate area + lateral wall of nasal cavity + lower eyelid with extended submental perforator flap combined with temporal muscle (JPG 6346 KB)

Supplementary file10 Figure 3a Radical surgery for the primary tumor (JPG 2696 KB)

Supplementary file11 Figure 3b Preset chin flap (JPG 3536 KB)

Supplementary file12 Figure 3c Repair of defect after resection of soft palate (JPG 2290 KB)

Supplementary file13 Figure 3d Submental flap was stitched and fixed to reshape the soft palate (JPG 2294 KB)

## Data Availability

All data generated or analysed during this study are included in this article. Further enquiries can be directed to the corresponding author.
