# Supplementary figures and images for: Application of prolonged submental perforator flap to repair the postoperative defect of upper airway malignancy
Source: Eur Arch Otorhinolaryngol. 2023 Aug 2;280(12):5507–18. doi: 10.1007/s00405-023-08131-5 (PMC10620253; doi:10.1007/s00405-023-08131-5)

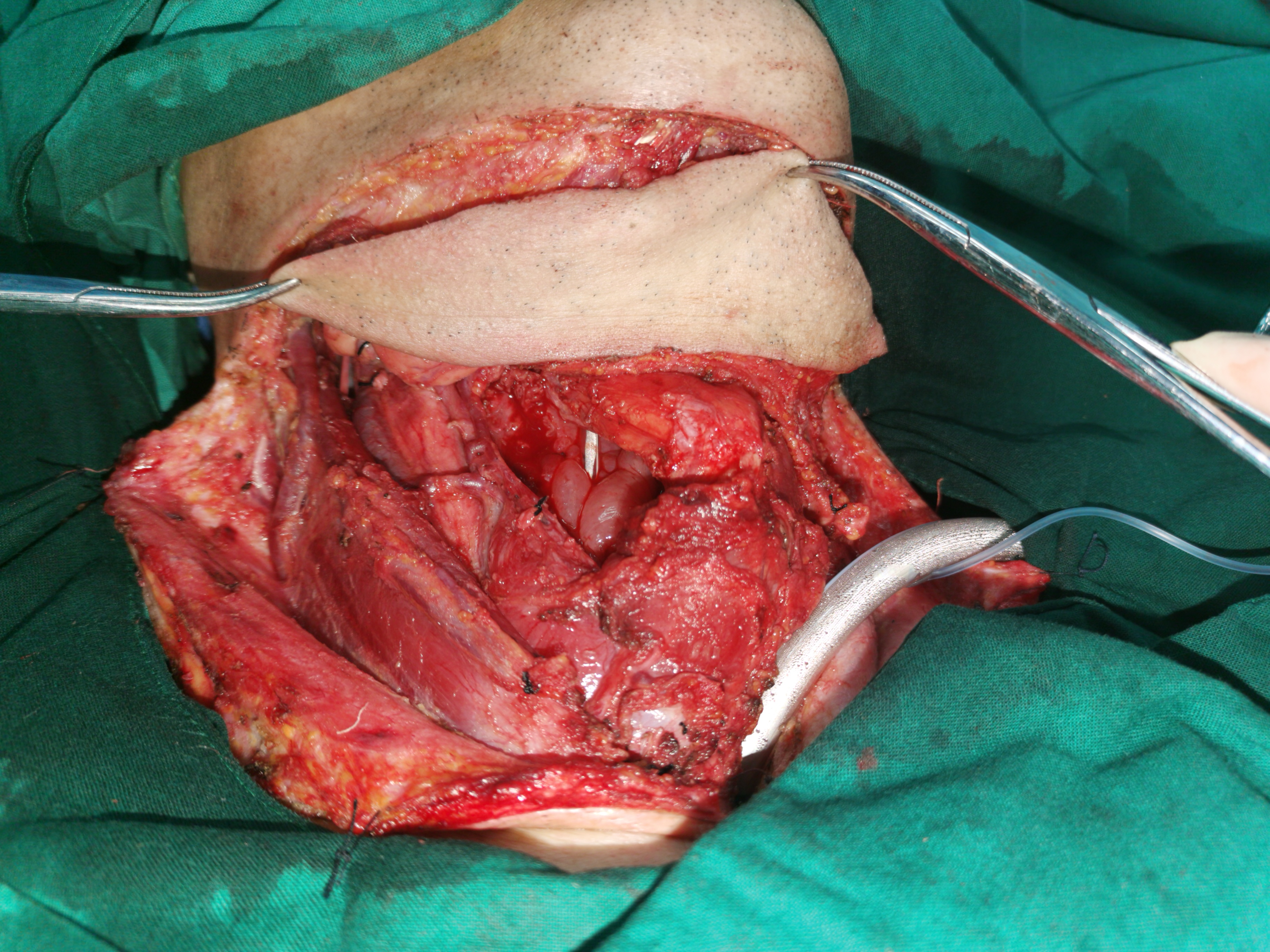

Supplement: Supplementary file 1 — Supplementary file1 Figure 1a Preset chin flap (JPG 2800 KB) [file 405_2023_8131_MOESM1_ESM.jpg]

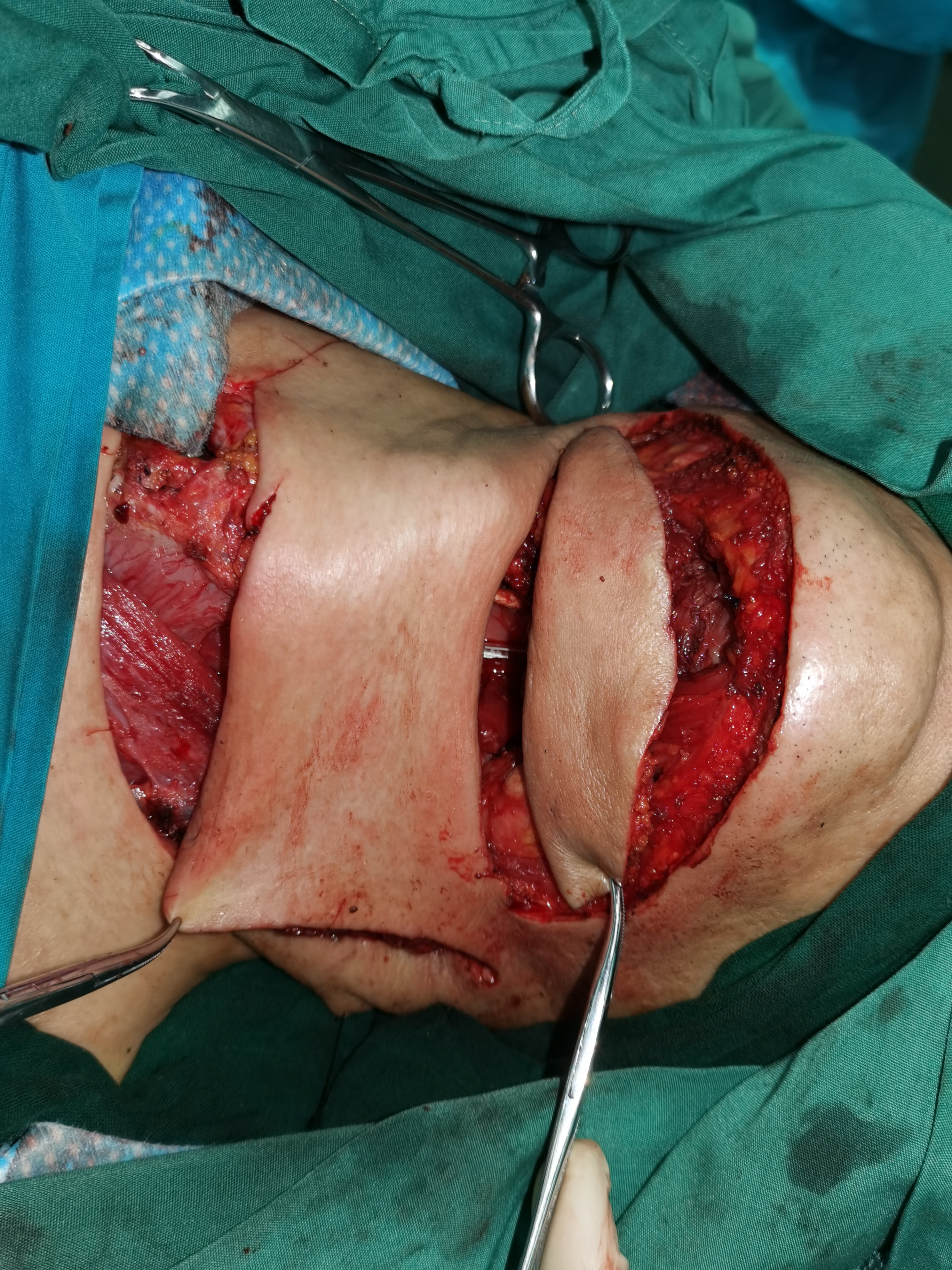

Supplement: Supplementary file 2 — Supplementary file2 Figure 1b Lateral incision (JPG 3531 KB) [file 405_2023_8131_MOESM2_ESM.jpg]

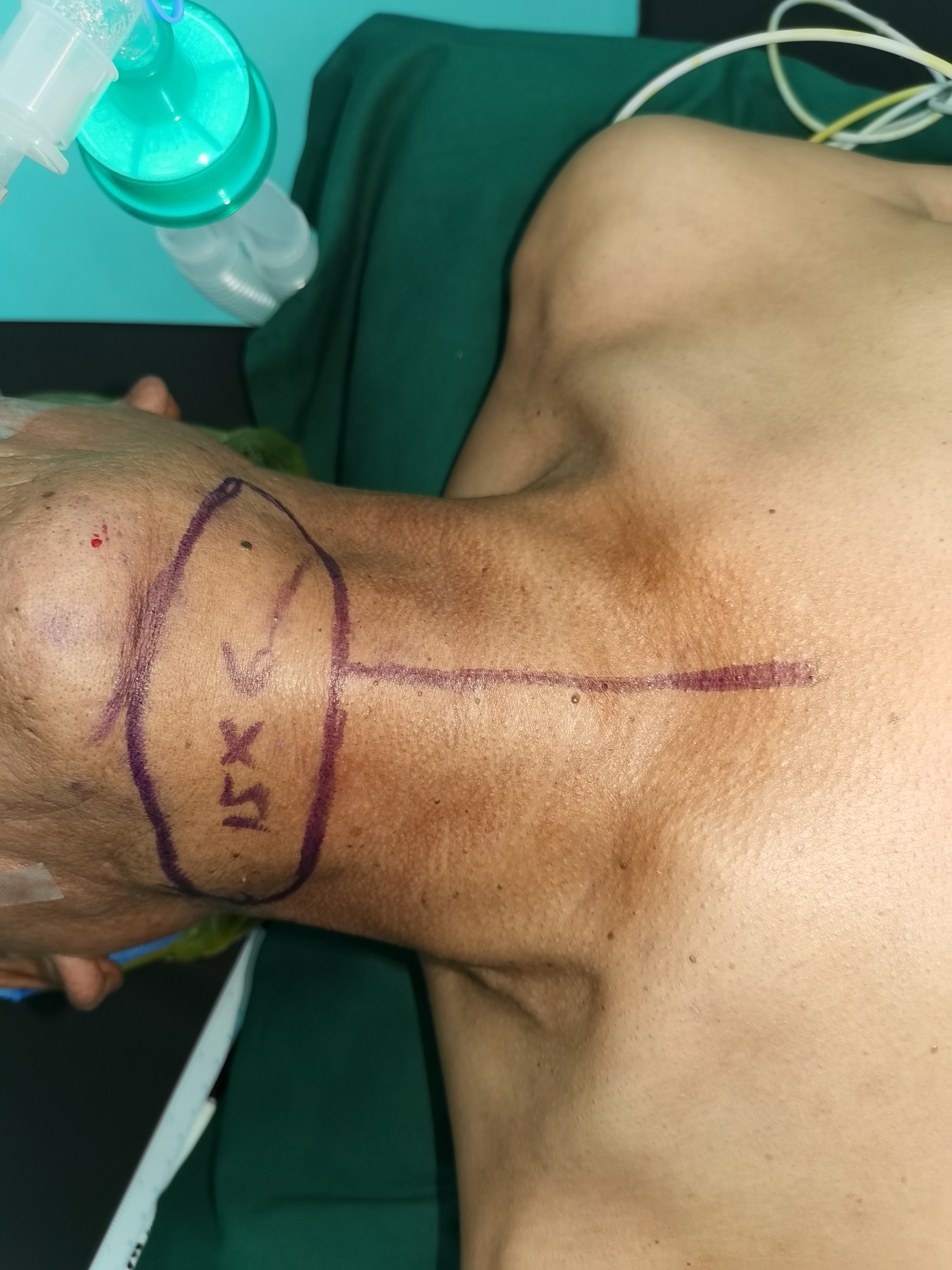

Supplement: Supplementary file 3 — Supplementary file3 Figure 1c T-shaped incision (JPG 2255 KB) [file 405_2023_8131_MOESM3_ESM.jpg]

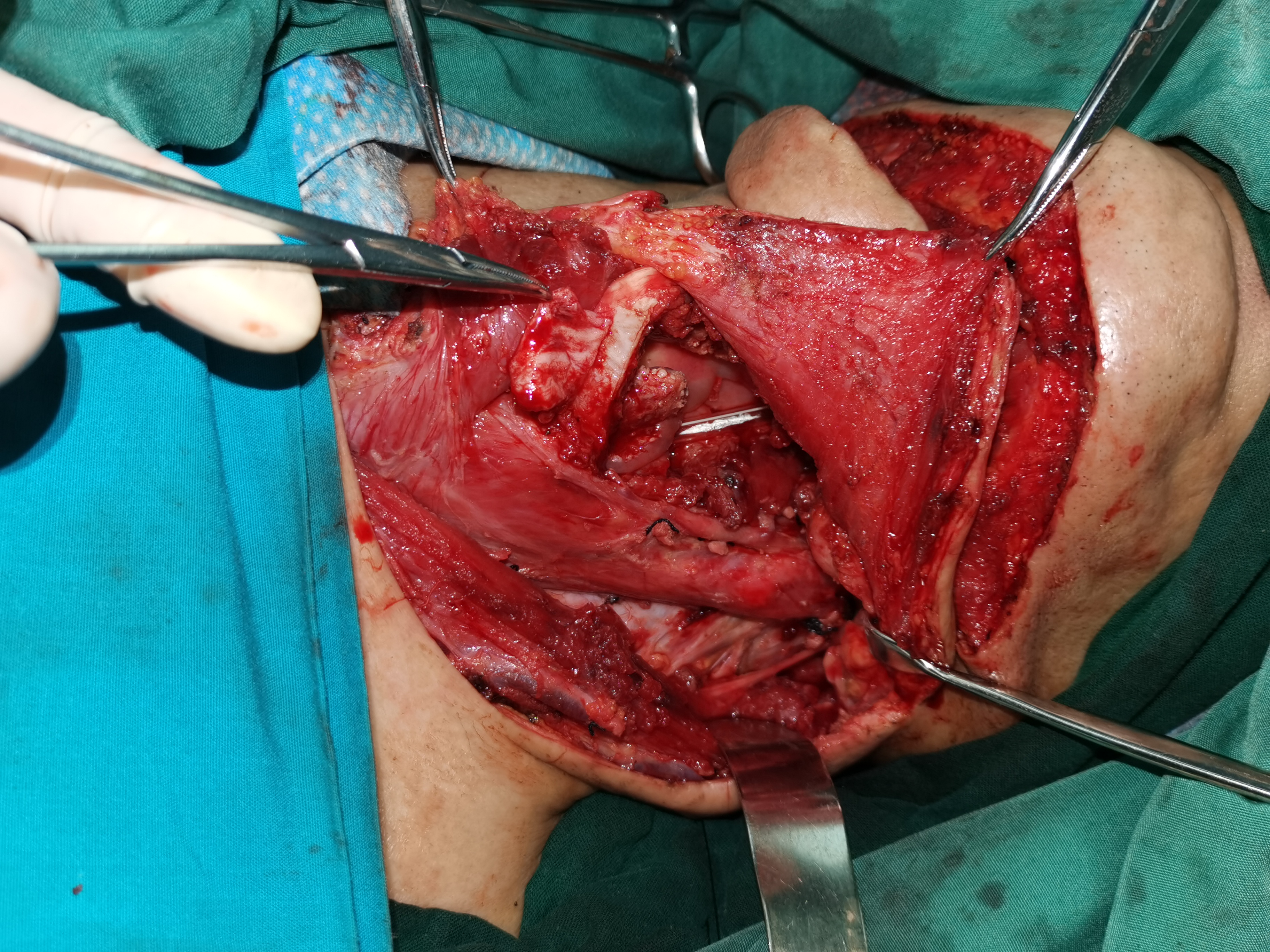

Supplement: Supplementary file 4 — Supplementary file4 Figure 1d Resected lesion (piriform fossa carcinoma) (JPG 3512 KB) [file 405_2023_8131_MOESM4_ESM.jpg]

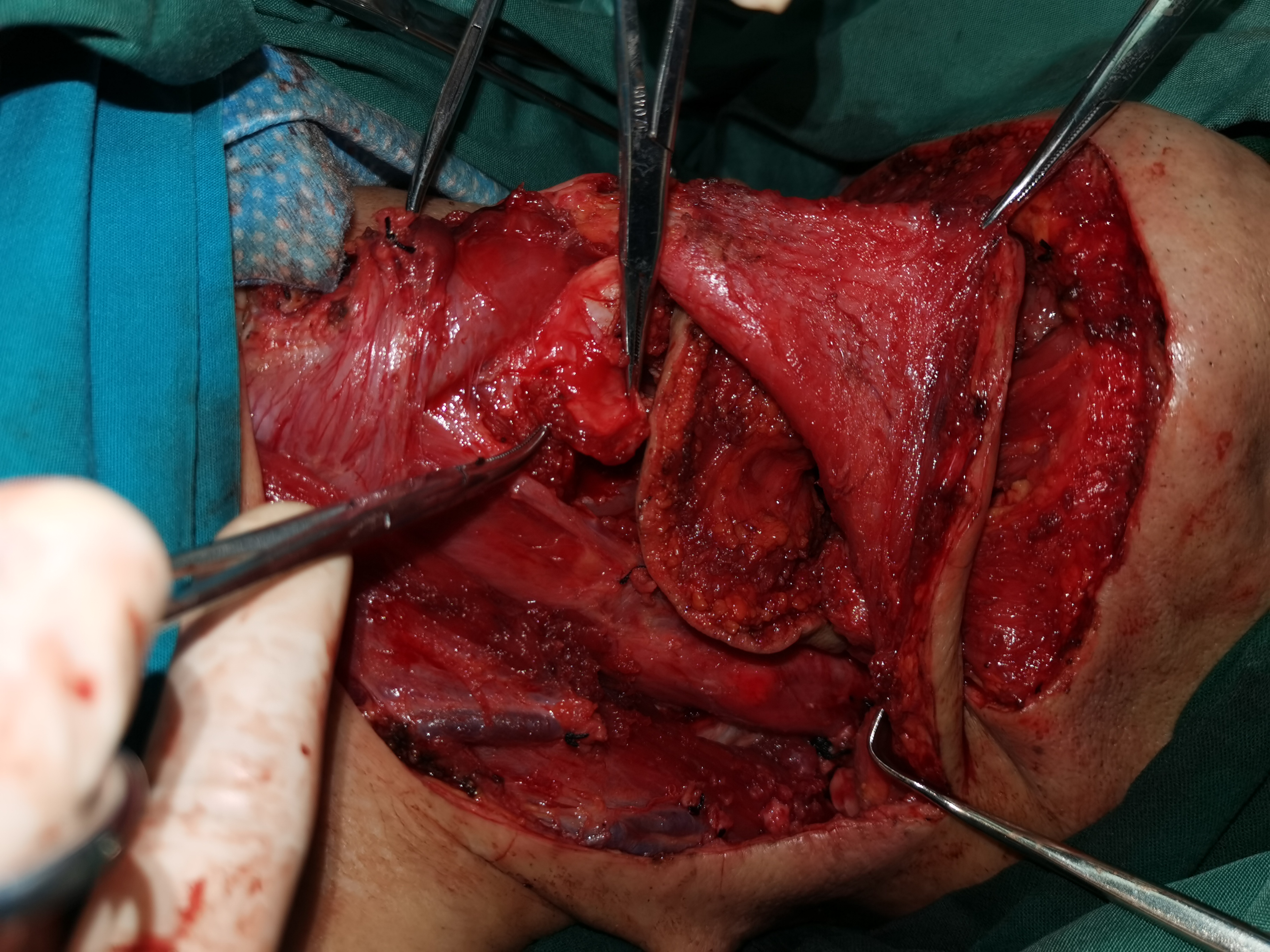

Supplement: Supplementary file 5 — Supplementary file5 Figure 1e Contralateral mental flap was used to repair the postoperative defect of the piriform fossa carcinoma (JPG 2574 KB) [file 405_2023_8131_MOESM5_ESM.jpg]

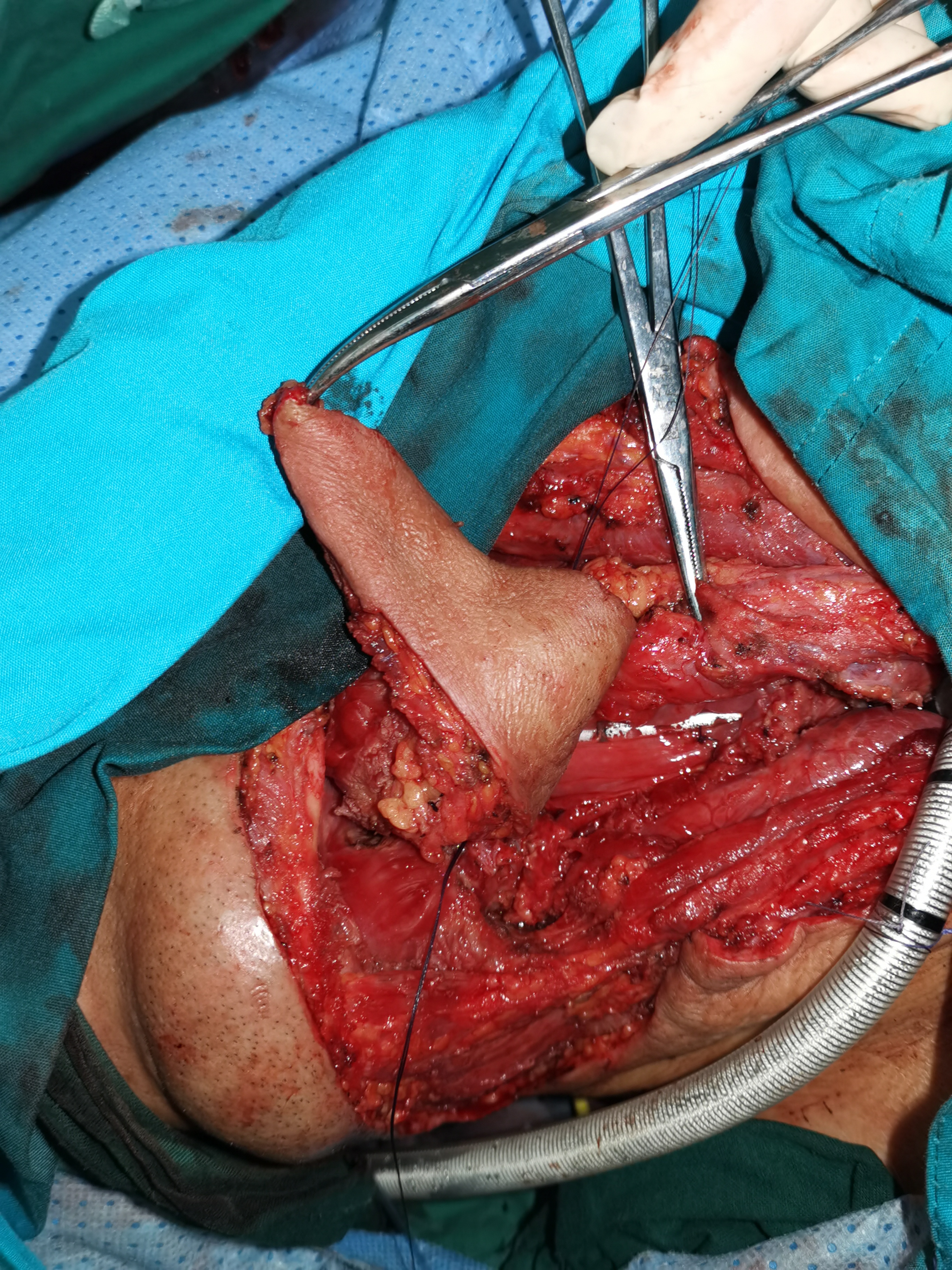

Supplement: Supplementary file 6 — Supplementary file6 Figure 1f Contralateral mental flap repair wound after total laryngectomy (retrocircumferential carcinoma) (JPG 3790 KB) [file 405_2023_8131_MOESM6_ESM.jpg]

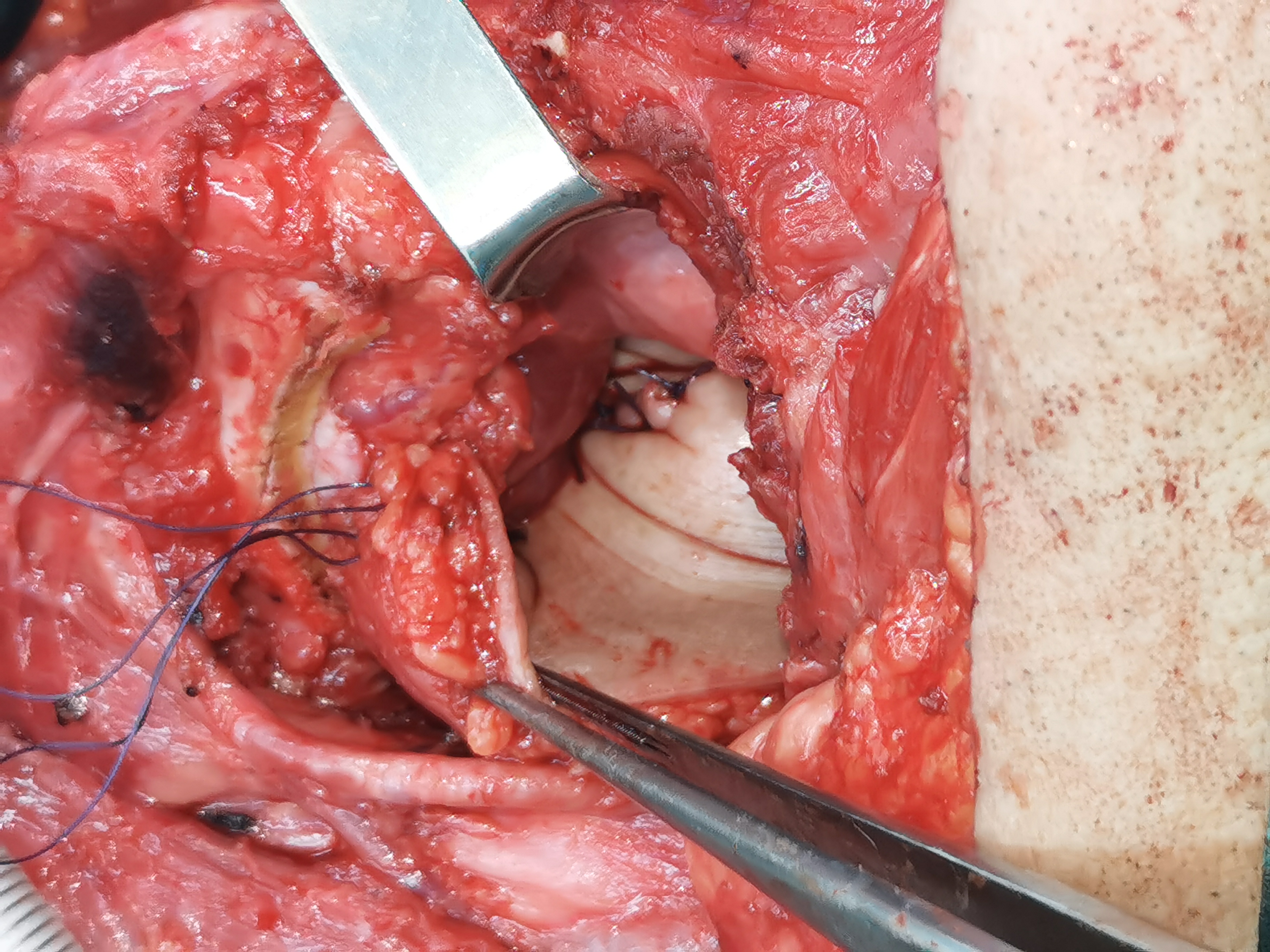

Supplement: Supplementary file 7 — Supplementary file7 Figure 1g Contralateral mental flap was repaired for posterior pharyngeal wall carcinoma, and the affected side of the piriform fossa was reconstructed (JPG 2469 KB) [file 405_2023_8131_MOESM7_ESM.jpg]

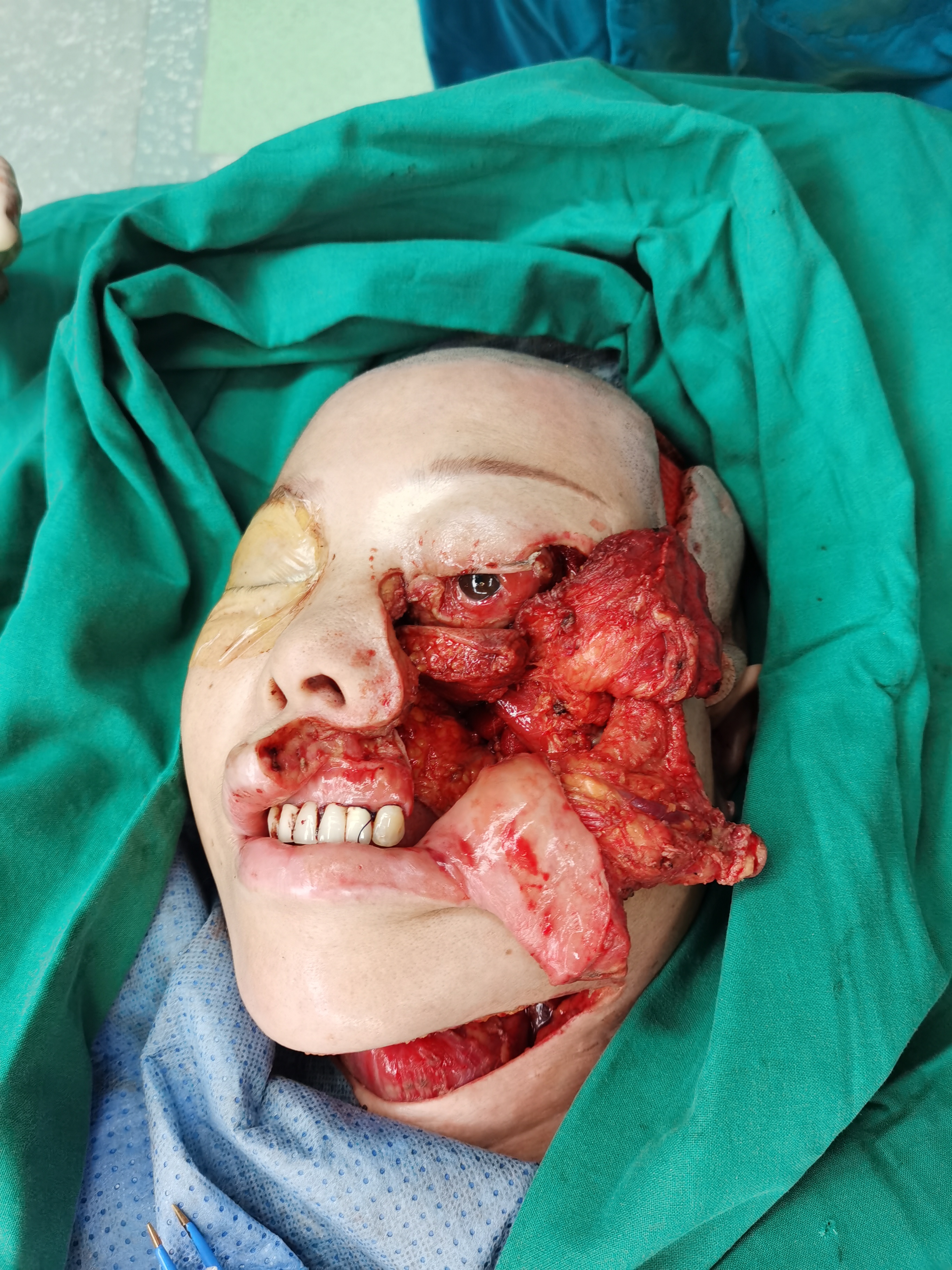

Supplement: Supplementary file 8 — Supplementary file8 Figure 2a Extended submental perforator flap to repair the defect after tumor resection (JPG 3149 KB) [file 405_2023_8131_MOESM8_ESM.jpg]

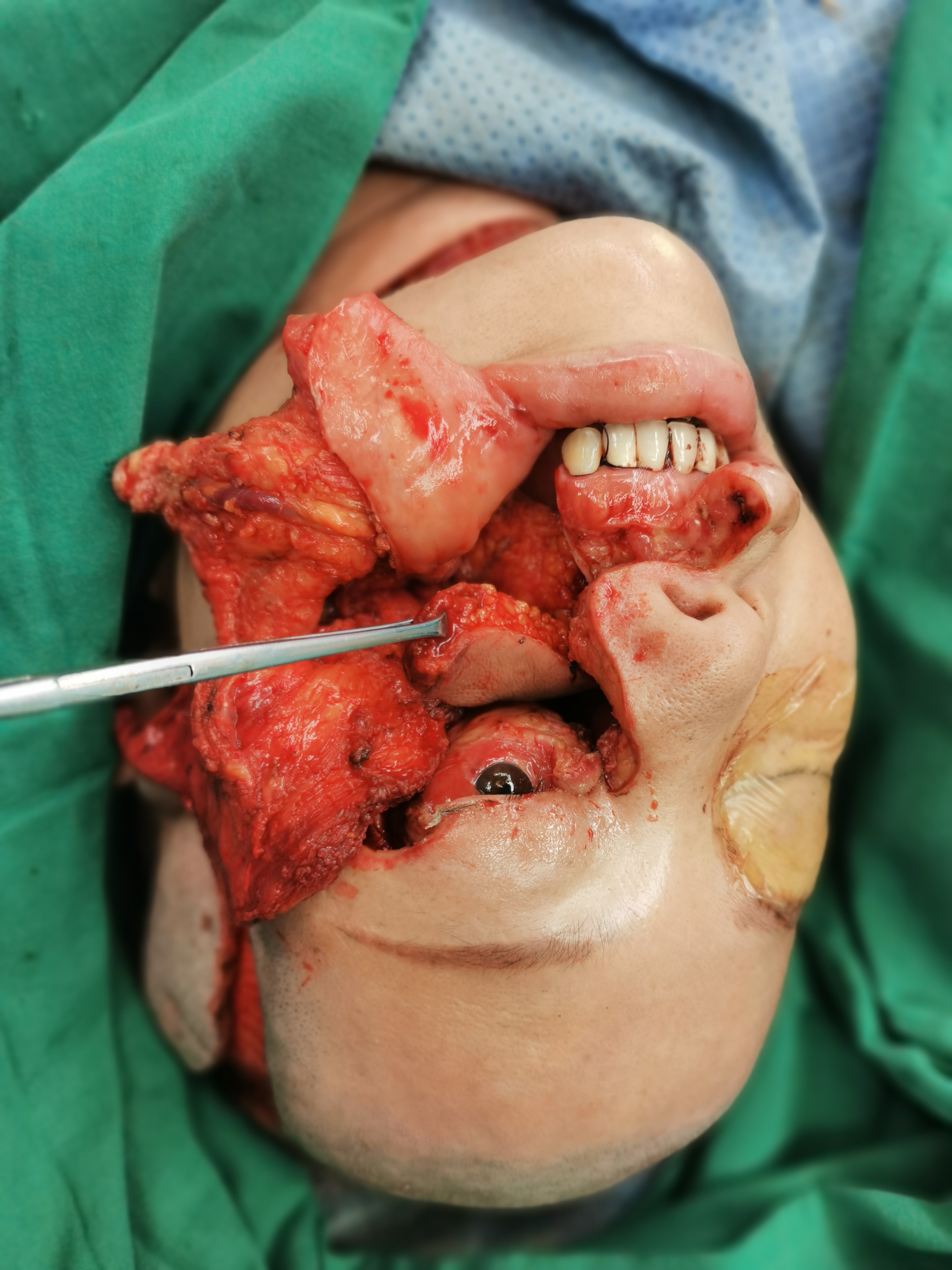

Supplement: Supplementary file 9 — Supplementary file9 Figure 2b Repair hard palate area + lateral wall of nasal cavity + lower eyelid with extended submental perforator flap combined with temporal muscle (JPG 6346 KB) [file 405_2023_8131_MOESM9_ESM.jpg]

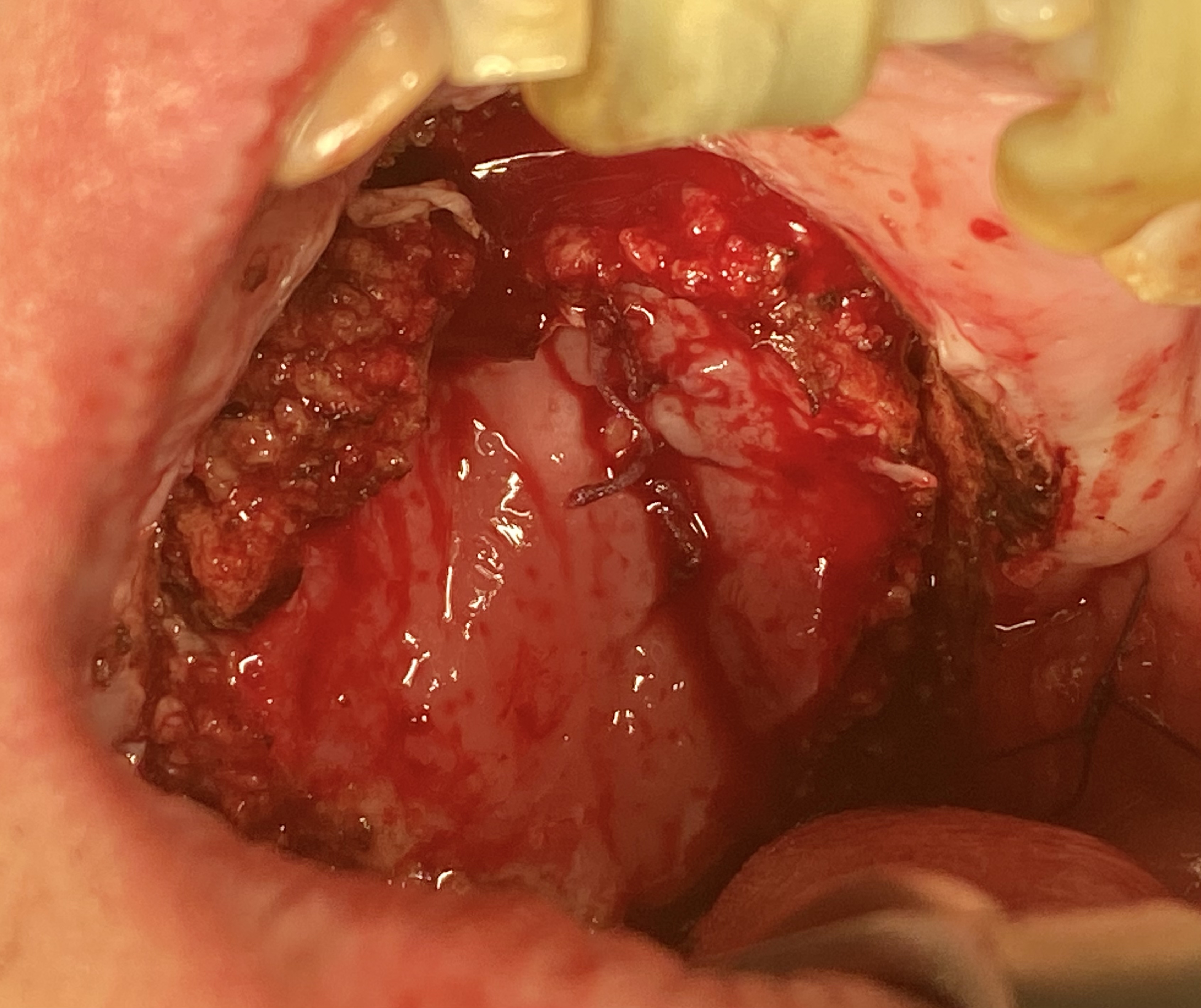

Supplement: Supplementary file 10 — Supplementary file10 Figure 3a Radical surgery for the primary tumor (JPG 2696 KB) [file 405_2023_8131_MOESM10_ESM.jpg]

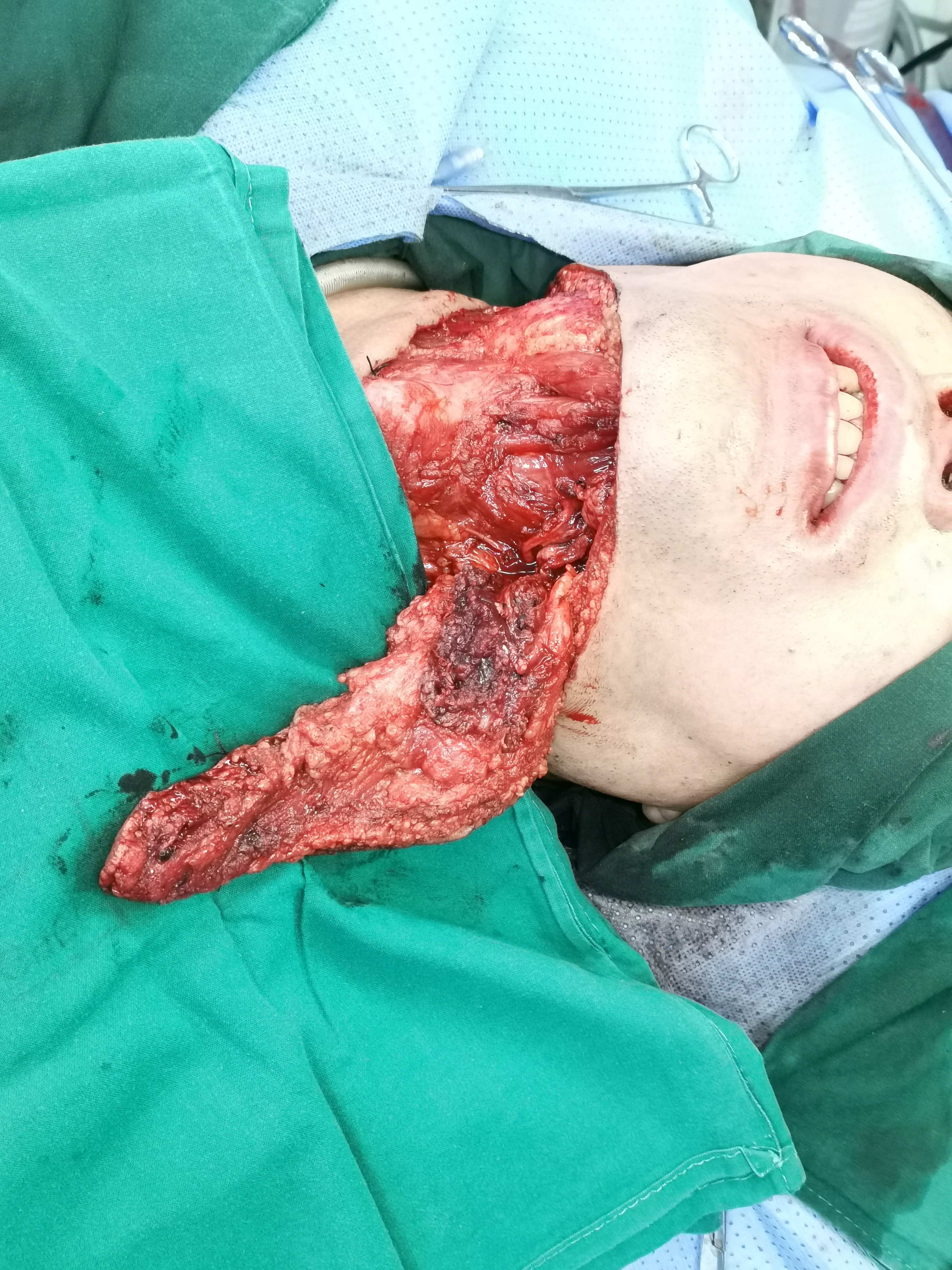

Supplement: Supplementary file 11 — Supplementary file11 Figure 3b Preset chin flap (JPG 3536 KB) [file 405_2023_8131_MOESM11_ESM.jpg]

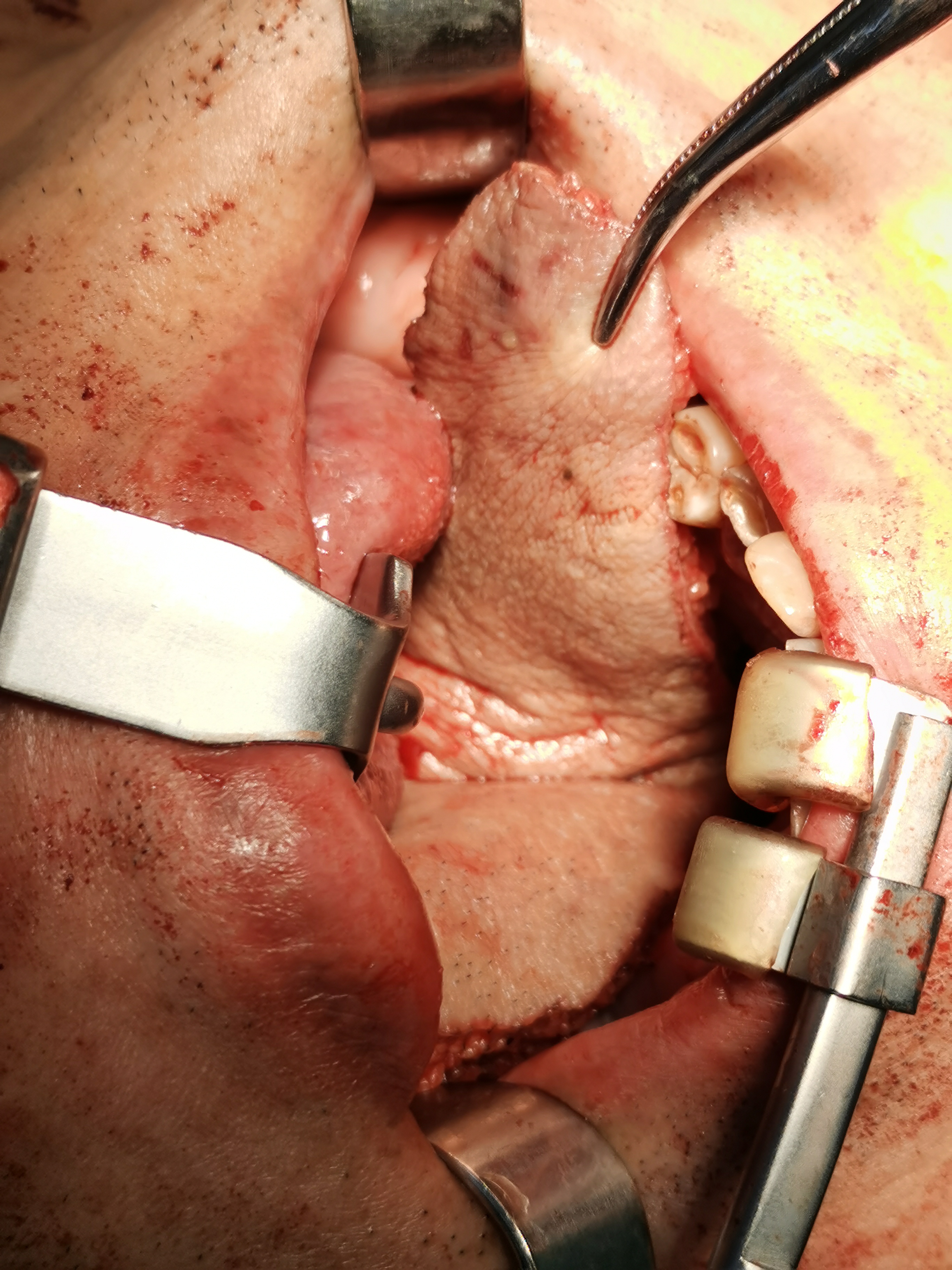

Supplement: Supplementary file 12 — Supplementary file12 Figure 3c Repair of defect after resection of soft palate (JPG 2290 KB) [file 405_2023_8131_MOESM12_ESM.jpg]

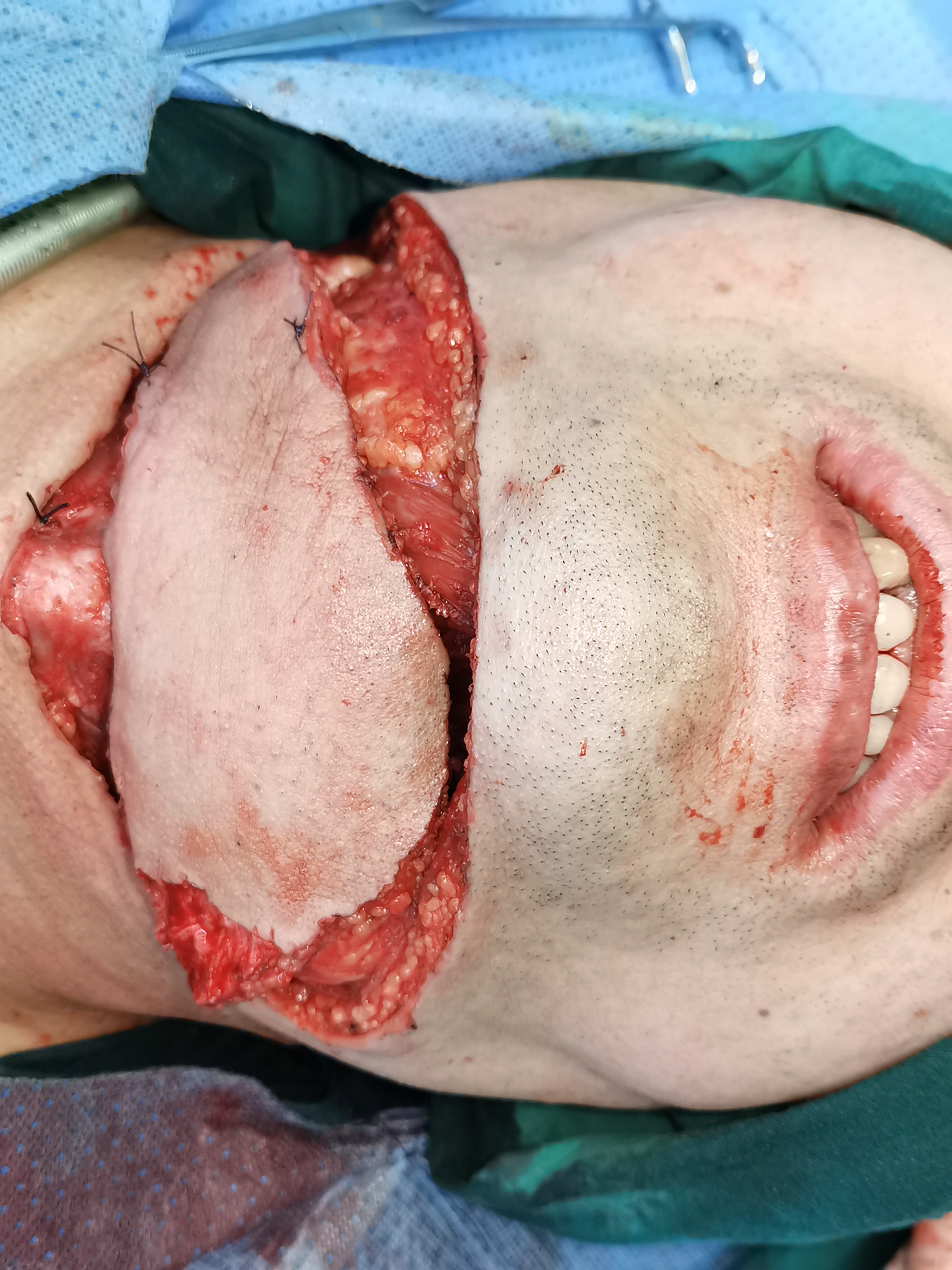

Supplement: Supplementary file 13 — Supplementary file13 Figure 3d Submental flap was stitched and fixed to reshape the soft palate (JPG 2294 KB) [file 405_2023_8131_MOESM13_ESM.jpg]
